# Synchrotron Near-Field Infrared Nanospectroscopy and Nanoimaging of Lithium Fluoride in Solid Electrolyte Interphases in Li-Ion Battery Anodes

**DOI:** 10.1021/acsnano.4c04333

**Published:** 2024-05-24

**Authors:** Andrew Dopilka, Jonathan M. Larson, Hyungyeon Cha, Robert Kostecki

**Affiliations:** † Energy Storage and Distributed Resources Division, Lawrence Berkeley National Laboratory, Berkeley, California 94720, United States; ‡ Department of Chemistry and Biochemistry, Baylor University, Waco, Texas 76798, United States; § Ulsan Advanced Energy Technology R&D Center, Korea Institute of Energy Research (KIER), Nam-gu Ulsan 44776, Republic of Korea

**Keywords:** LiF, anode, SEI, interface, interphase, nano-FTIR, SINS

## Abstract

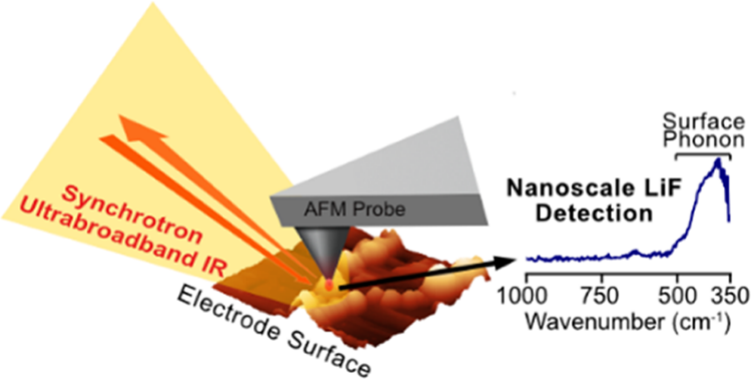

Lithium fluoride (LiF) is a ubiquitous component in the solid electrolyte interphase (SEI) layer in Li-ion batteries. However, its nanoscale structure, morphology, and topology, important factors for understanding LiF and SEI film functionality, including electrode passivity, are often unknown due to limitations in spatial resolution of common characterization techniques. Ultrabroadband near-field synchrotron infrared nanospectroscopy (SINS) enables such detection and mapping of LiF in SEI layers in the far-infrared region down to ca. 322 cm^–1^ with a nanoscale spatial resolution of ca. 20 nm. The surface sensitivity of SINS and the large infrared absorption cross section of LiF, which can support local surface phonons under certain circumstances, enabled characterization of model LiF samples of varying structure, thickness, surface roughness, and degree of crystallinity, as confirmed by atomic force microscopy, attenuated total reflectance FTIR, SINS, X-ray photoelectron spectroscopy, high-angle annular dark-field, and scanning transmission electron microscopy. Enabled by this approach, LiF within SEI films formed on Cu, Si, and metallic glass Si_40_Al_50_Fe_10_ electrodes was detected and characterized. The nanoscale morphologies and topologies of LiF in these SEI layers were evaluated to gain insights into LiF nucleation, growth, and the resulting nuances in the electrode surface passivity.

## 1 Introduction

To improve Li-ion battery performance, intermetallic anodes have been extensively researched due to their higher gravimetric and volumetric capacities.^1^ Among the intermetallics, Si-based anodes can have 10 times the gravimetric capacity than the standard graphite electrode and thus represent a promising path for improving the energy density of current Li-ion batteries.^2^ While the challenges associated with the large volume change and mechanical stability have been addressed extensively in the past decade, the calendar life of Si-based cells has been largely overlooked and now represents a major roadblock as Si materials near commercial adoption.^3^ At the heart of the calendar life fade is a poorly passivating solid electrolyte interphase (SEI) layer on the Si anode surface, which continually consumes the electrolyte and Li inventory, resulting in an impedance increase and capacity fade during rest.^3,4^

The SEI is a passivation layer that forms at the anode of Li-ion batteries and determines cycle/calendar life, rate performance, and safety.^5^ This 10′s-of-nanometers thin layer is comprised of various decomposition products of the liquid electrolyte, which collectively passivate the electrode surface and its properties are very sensitive to formation and aging conditions.^5^ An SEI successfully passivates an electrode surface when parasitic reactions with the electrolyte are minimized to a point that enables a sufficient lifetime of the Li-ion battery. Despite the important role the SEI plays in Li-ion battery performance, its exact structure, composition, and function are still an open question and an area of active research.^6^ For Si-based anodes, the challenge lies in understanding how the small nanoscale alterations in the initially formed passivation layer relate to the stability during the calendar life aging. Because there is no volume expansion or cycling during this aging, there must be a passive chemical or electrochemical discharge occurring that leads to gradual electrolyte decomposition and accumulation of insoluble and soluble reaction products that result in reduced performance, i.e., capacity loss or increasing impedance. Some hypotheses about the origin of these parasitic reactions include continual HF production, SEI dissolution and regrowth, and the higher reactivity of the Si and Li_*x*_Si surface.^3^ To mitigate these issues, various strategies have been developed such as electrolyte additives, surface coatings, and active material modification.^3^

**Figure 1:**
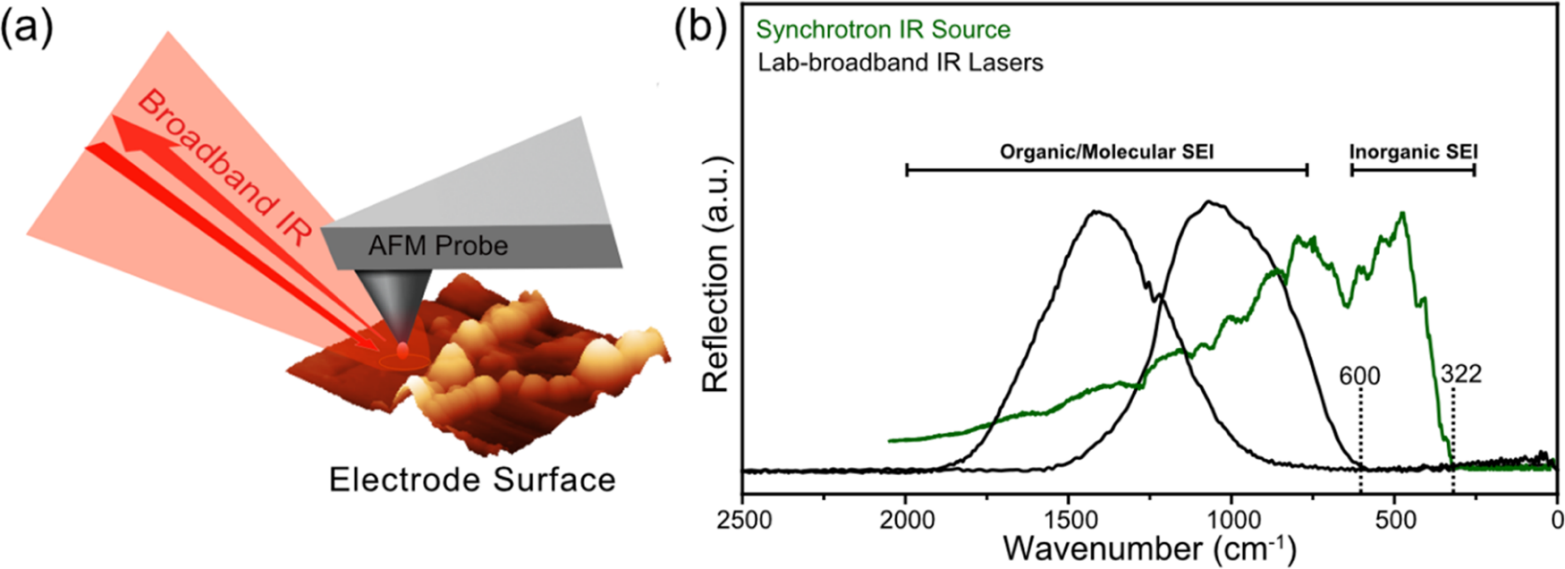
(a) Schematic representation of the operating principle of nano-FTIR spectroscopy. (b) Comparison of the nano-FTIR reflection of different broadband IR sources taken on a Si wafer.

LiF is a notable electrolyte decomposition product due to its ubiquitous presence in the SEI of fluorine-containing electrolytes and is often attributed to being essential for improved passivation of Li-ion anodes.^7−10^ LiF, in its bulk crystalline form, is resistive to Li-ion conduction^11,12^ but can have higher conductivity if disordered or nanocrystalline;^13,14^ therefore, the size and distribution of LiF are relevant parameters for SEI function and operation. LiF can form in a variety of pathways from the decomposition of the fluorine-containing molecules in the electrolyte, which affects its resulting morphology and its role in passivating the interface. For instance, it has been demonstrated that H_2_O impurities in LiPF_6_-based electrolytes result in the formation of HF due to the hydrolysis of the PF_5_ molecule.^15−17^ The resulting HF can electrocatalytically react on the surface of metal electrodes to form H_2_ gas and LiF deposit on the electrode surface at a rate that is dependent on potential, electrode surface composition, and HF concentration.^18,19^ Additionally, the PF_6_^–^ molecule can directly react at the electrode surface to form LiF, as forming solid LiF is very energetically favorable.^18^ Another factor influencing LiF formation and resulting particle morphology is its solubility within the electrolyte itself, which could affect crystal growth over long periods of time.^20,21^ It can be also formed via the decomposition of fluorine-based additives such as fluoroethylene carbonate, which has been shown to beneficially modify the SEI layer’s structure.^22^ Despite LiF’s widespread presence in the SEI of Li-ion anodes, there are still unknowns about how to control its formation and resulting topology and how this affects surface passivation, especially in the context of the cell calendar life.

**Figure 2:**
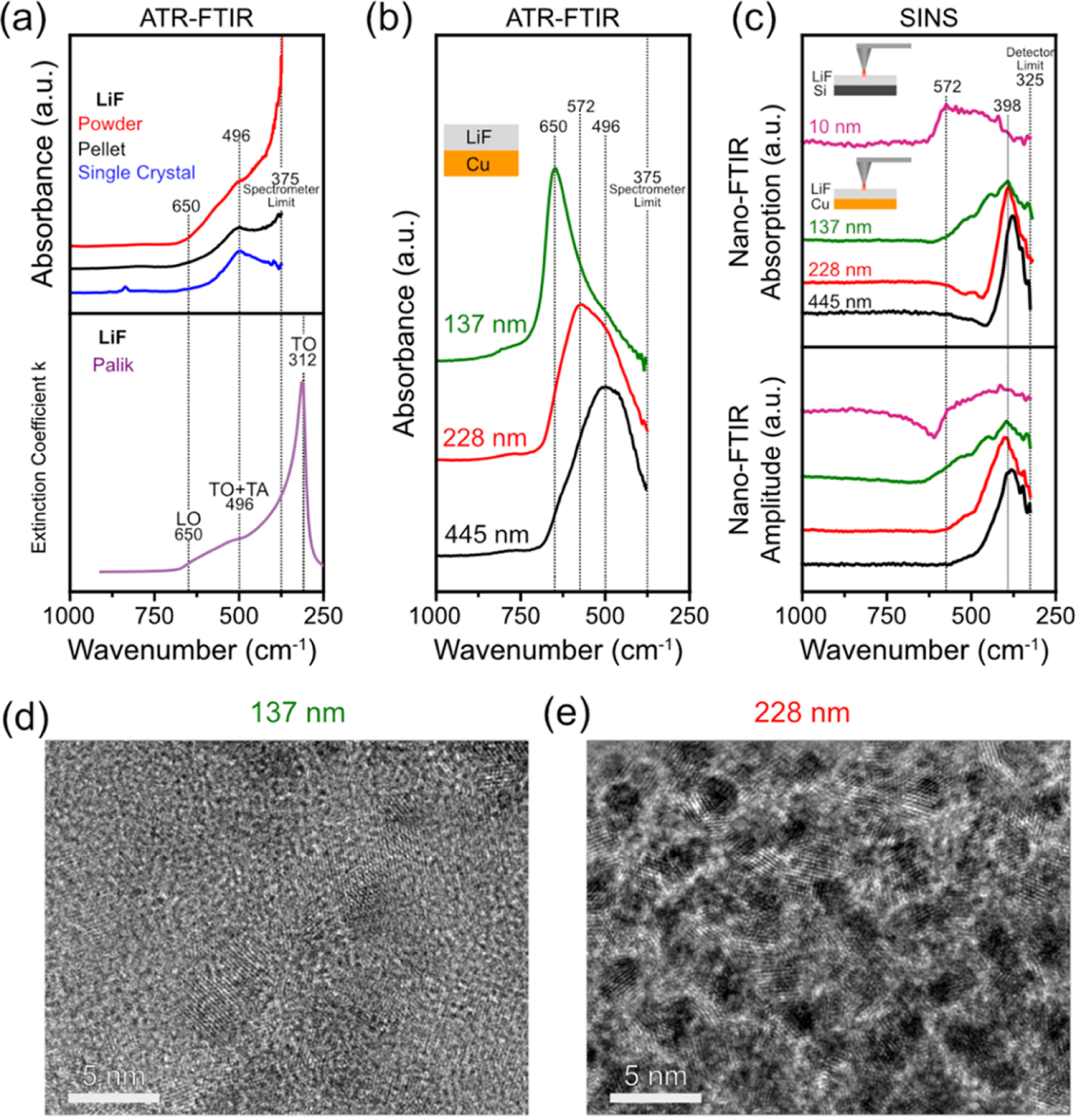
(a) ATR-FTIR spectra of LiF powder, pellet, and single crystal along with the extinction coefficient as a function of the wavenumber from Palik.^66^ (b) ATR-FTIR spectra of evaporated thin films of LiF of different thicknesses. (c) SINS absorption and amplitude spectra of evaporated LiF thin films of different thicknesses. HRTEM images of the evaporated LiF thin film with a thickness of (d) 137 nm and (e) 228 nm.

The primary challenge of determining the relationships of the SEI’s function and operation to its components (i.e., LiF) is detection, characterization, and 3-D mapping of buried, nanometer-scale film components, which are very sensitive to handling conditions and often have similar spectral signatures as the electrolyte. Techniques such as X-ray photoelectron spectroscopy (XPS),^23^ Fourier transform infrared spectroscopy (FTIR),^24^ Raman spectroscopy,^25^ cryogenic transmission electron microscopy (cryo-TEM),^26,27^ time-of-flight secondary ion mass spectroscopy (TOF-SIMS),^28^ and many other techniques have been widely used to study the SEI layer due to their high chemical and/or structural sensitivity. Most notably, cryo-TEM imaging that combines nanometer-scale spatial resolution with atomic structure sensitivity provided insights into the makeup of the SEI layer.^26,27^ However, there is still a significant challenge to characterize the SEI without altering it from its operational state as it can be easily damaged by air/vacuum exposure,^29^ electron, X-ray or optical beam damage,^29^ and washing procedures to remove the residual electrolyte.^30,31^ Careful environmental control during sample processing and characterization measurements is essential to obtain meaningful results from the aforementioned techniques.

FTIR and Raman spectroscopy are nondestructive, low-energy photon optical techniques that can be applied under ambient conditions with high definition with regard to the specific vibrational modes of crystalline lattices and molecular structures. FTIR spectroscopy, which is primarily used for the characterization of organic and molecular compounds in the SEI layer,^32^ has also been used to identify Li-based inorganic phases but to a much lesser extent.^33,34^ In fact, phases such as LiF, Li_2_O, and LiH, which are identified as important SEI components,^7,34−100^ are infrared active with their fundamental vibrational modes in the range from 550 to 312 cm^–1^.^36−38^ However, despite FTIR’s ability to detect a variety of organic and inorganic SEI components, the spatial resolution is limited by the wavelength of the incident light and by the numerical apertures of the condenser and objective lens systems (so-called diffraction limit), for which IR light limits the probing spatial resolution to a few micrometers. Such a relatively low spatial resolution is inadequate for the detection and imaging of individual building blocks in the SEI layer, whose size typically does not exceed 10–20 nm.

The need for an imaging technique that retains the various contrast mechanisms of standard optical microscopy methods while attaining spatial resolution beyond the diffraction limit challenge led to the development of several near-field scanning optical probes in the past decades.^39,40^ In particular, scattering scanning near-field optical microscopy (s-SNOM)^40^ and the related nano-FTIR^41^ spectroscopy have been applied in recent years to probe material’s local properties at nanometer resolution (∼20 nm) in various applications such as catalysis,^42^ electronics,^43^ photonics,^44^ biology,^45−47^ and electrical energy storage.^48,49^ This technique involves using a metalized atomic force microscopy (AFM) probe, which serves as a local optical near-field IR antenna to enhance the electromagnetic field at the tip’s end (Figure 1a). The induced “near-field” evanescent wave couples with the IR active vibrational modes in the sample and modifies the amplitude and phase of the backscattered light, which is then collected by the detector.^39,50^ Since the magnitude of the near-field enhancement is nonlinearly dependent on the tip/sample distance, the near-field interaction can be separated from the large far-field background by operating in constant tapping mode and subsequent signal demodulation to higher harmonics with lock-in amplification at the tapping frequency.^41,51^ The result is the extraction of the near-field optical response of the sample with the probing area similar to the tip radius (ca. 20 nm) and probing depth dependent on the sample’s optical properties. Subsurface detection has been demonstrated to be around 100 nm through insulating materials,^52,53^ whereas materials with high electronic conductivity (e.g., metals) attenuate the near-field within a few nanometers from the surface. While s-SNOM refers to imaging at a single wavelength, nano-FTIR involves illuminating the tip/sample interface with a broadband IR source and processing the backscattered light in a similar manner as traditional FTIR, resulting in nanoscale IR absorption spectra.^41^ Like the standard far-field FTIR, nano-FTIR is nondestructive and can be applied in ambient conditions, which ensures that the sensitive SEI layer remains undisturbed during the measurement.

LiF is widely believed to be a determining factor for the effective passivation of Li-ion anodes;^7−10^ however, its structure, topology, and distribution in the film are rarely investigated due to the lack of suitable characterization techniques. XPS is the most common way to quantify and detect the presence of LiF in the SEI layer,^29,54,55^ but the large probing area (10′s of microns) prevents accurate nanometer-scale mapping to support conclusions about the role that LiF plays in the SEI’s function and operation. For instance, Huang et al. did a comparative characterization of the SEI on Li metal plated on Cu with XPS and cryo-TEM.^21^ The authors found that with cryo-TEM, the presence of large 100–200 nm LiF particles scattered over the Cu surface was observed, whereas the Li metal wires were covered with a thin amorphous SEI layer, suggesting that LiF was not actively passivating the Li metal. In contrast to the cryo-TEM images, the XPS results are unable to assess this nuanced view of the spatial distribution of the LiF on the nanometer scale, which is relevant for its function in the SEI layer. Therefore, there is a need to develop additional characterization methods and experimental approaches capable of probing the SEI structure with adequate spatial resolution in a nondestructive manner to make a direct connection between the SEI’s nanostructured morphology and its passivation performance.

We have been applying s-SNOM and nano-FTIR to study electrode surface species in a variety of systems, including the SEI layer on Li-ion electrodes.^30,48,56−59^ We deployed a nano-FTIR study to investigate the SEI layer formed on thin-film amorphous Si films to gain insights into the SEI layer dependence on the passivation performance. We found that the surface of the Si electrode after lithiation was covered uniformly by organic decomposition products such as lithium ethylene decarbonate (LiEDC) and poly(ethylene oxide) (PEO) in addition to salt decomposition products originating from the PF_6_^–^ molecule in addition to LiF (detected with XPS).^30^ However, the probing and mapping of Li-inorganic phases with nano-FTIR present a challenge because their primary vibrational modes reside at low energies, i.e., 550–312 cm^–1^,^36−38^ which is out of range of quantum cascade IR lasers (QCL) and broadband difference frequency generator (DFG) lasers (600–3000 cm^–1^) used in standard benchtop nano-FTIR spectrometers (Figure 1b).

Synchrotron broadband IR sources (50,000–10 cm^–1^) have been implemented into nano-FTIR systems, which are termed synchrotron infrared nanospectroscopy (SINS).^60−62^ A liquid He-cooled Ge:Cu detector along with a synchrotron IR source^62^ has enough spectral range and sensitivity in the far-IR (<322 cm^–1^) (Figure 1b) to detect and characterize vibration modes of LiF and other inorganic Li phases as well as organic/molecular components of the SEI region at higher energies <2000 cm^–1^.

In this work, we use ATR-FTIR to characterize a series of LiF bulk and thin-film reference samples to determine a baseline of LiF structural characteristics compatible with SINS measurements. Then, the LiF structure and distribution were evaluated with SINS on the nanometer scale in the SEI layer on a Cu- and Si-based model Li-ion electrodes in 1.2 M LiPF_6_ in an EC/EMC 3:7 wt % electrolyte. Variations of LiF structural and topological characteristics are then correlated to the different degrees of surface passivity observed for these Li-ion anodes in organic carbonate electrolytes.

## 2 Results and Discussion

### 2.1 Characterization of LiF Model Samples with ATR-FTIR, SINS, and TEM

LiF model samples were investigated to determine the effect of structure, thickness, surface roughness, and degree of crystallinity on the infrared absorption spectrum of LiF. The ATR-FTIR spectrum of LiF powder (Figure 2a, **red**) shows two broad absorption features. One at ca. 496 cm^–1^, and a second, very large feature whose onset nears the low-energy limit of the IR detector (375 cm^–1^). The former feature has been attributed to a two-phonon absorption involving the bulk transverse optical (TO) mode and the transverse acoustic (TA) mode.^63,64^ While the lower-energy side of the later absorption feature was not obtained—due to the cutoff of our IR detector—this prominent feature is assigned to the well-known bulk transverse optical (TO) mode of LiF expected at ca. 312 cm^–1^, which arises from Li^+^ and F^–^ ions moving 180 deg out of phase with each other.^37,65^ For clarity, the extinction coefficient of crystalline LiF, as reported by Palik,^66^ is provided (Figure 2a, **bottom, purple**), which clearly displays a similar large peak centered at 312 cm^–1^. As expected, there is no peak for the bulk longitudinal optical (LO) mode at ca. 650 cm^–1^, which is infrared inactive unless special circumstances occur.^67^

A pressed and polished LiF pellet (Figure 2a, **black**) showed a similar infrared absorption spectrum, albeit with bandwidth narrowing of the peak at 496 cm^–1^ and a less pronounced slope onset for the TO mode, again from bandwidth narrowing. This narrowing is likely due to a more robust contact between the diamond ATR crystal and the LiF sample made possible by the pressed and polished pellet having a relatively smoother surface. A spectrum of single-crystal LiF continues this trend (Figure 2a, **blue**). We note that in all of these measurements, the thickness of the LiF samples was well beyond the nominal ATR-FTIR diamond crystal probe penetration depth of ca. 2 μm.^68^ Thus, in theory, the only interface exposed to probing was the LiF/diamond interface. However, in the case of the LiF powder, we expect a larger contribution from the LiF/N_2_ gap interface due to the higher roughness and less packing efficiency of LiF particles, resulting in void space. The difference between the spectra from the powder and pellet samples can be rationalized by considering the absorption from surface phonon polaritons (as opposed to purely bulk absorption), which emerge between the bulk LO and TO modes,^69,70^ i.e., between 667 and 312 cm^–1^, which effectively broaden the absorption range. This can occur, and has been observed by others,^71^ when nano-to-microscale regions of the ATR crystal/sample interface are imperfect, containing air gaps or, in the case of size effects, when particles are smaller or similar to the wavelength of interacting light.^72^ This would refer to the presence of local N_2_ gaps, resulting in LiF/N_2_ interfaces amenable to support surface phonons, and considering that the real part of the LiF dielectric constant close to −1^73^ for single-crystal LiF (see Palik^66^) would produce a vibration band at ca. 571 cm^–1^.

While bulk material spectral characteristics are often used as a reference to experimental SEI spectra, composition, size and shape of interfaces, and type of the characterization probe can significantly alter the observed infrared spectra in various ways, especially via the activation of surface modes.^67,72^ Therefore, bulk LiF (powder or pellet) where the N_2_ gap between the probe and LiF allows surface phonons to couple to the IR light may not be a suitable reference for SINS of LiF in the SEI, and instead, a thin-film model sample would be more appropriate to mimic the SEI, which is typically a few 10′s of nanometers thick.

Three thin-film LiF model samples were fabricated and characterized. FIB-SEM was used to determine the thickness of the evaporated LiF films at 137, 228, and 445 nm (Figure S1), and HAADF and STEM EDS were used to validate the elemental composition of the LiF layer (Figure S2). XPS measurements of the evaporated films confirmed the elemental purity of the deposited LiF (Figures S3–S4). AFM topography images of the thin films show low RMS surface roughness (4.6 nm for 228 nm thick LiF), meaning that surface roughness effects on the IR spectra should be minimal (Figure S5).

Figure 2b shows the ATR-FTIR spectra of the LiF thin films, which vary significantly with the film thickness. The 137 nm film spectrum shows a strong peak at ca. 650 cm^–1^, which matches with the reported LO mode of LiF (which is typically infrared inactive), whereas the spectrum of the 445 nm film displays features similar to the bulk LiF sample: a broad peak at 496 cm^–1^ and only a weak shoulder at 650 cm^–1^. These observations are centrally related to the penetration depth of the ATR-FTIR evanescent wave, which for a diamond ATR crystal and LiF can easily reach ca. 2 μm.^68^ For the 137 nm film, IR light can readily reach the LiF/Cu interface and excite the LO mode by either (i) plasmon–phonon coupling at the dielectric/metal interface^74−77^ or by (ii) the Berreman effect, which involves the activation of surface modes at the LO frequency in thin films on a metallic substrate at oblique angles of illumination.^78,79^ The Berreman effect can be further influenced by the refraction index of the overlayer, the angle of incidence of the incoming light, and the substrate material.^67^ As the LiF film thickness increases and the IR excitation of the LiF/Cu interface diminishes, the absorption spectra trend toward the bulk characteristics, revealing TO + TA modes at 572 and 496 cm^–1^ between the bulk LO and TO modes. These measurements acutely highlight that many factors determine the spectra for thin films, not least of which are how many interfaces are being probed, the thickness/morphology/spatial extent of the material of interest, and if (perhaps unexpectedly) surface resonances can arise. All this emphasizes the need for careful, well-controlled model reference systems to characterize prior to delving into complex, multicomponent systems, like an SEI.

The SINS local single point spectra (Figure 2c) of the LiF thin films show a striking difference from the average ATR-FTIR spectra of the same samples, pointing to the local and shallow SINS probing depth, and the likelihood of observing surface phonons due to the N_2_ gap present between the sample and the metallic probe. The SINS absorption and amplitude spectra of the 445 nm LiF film show a sharp peak at 379 cm^–1^. This apparent shift of the TO mode from 312 cm^–1^ for bulk LiF to higher energies is a common observation in the SINS spectra of strong oscillators. It originates from the strong tip–sample coupling of the surface phonon polaritons at frequencies where the real part of the dielectric function is slightly negative, resulting in enhancement of the near-field.^40,80−84^ This phenomenon can be rationalized by considering that strong oscillators have negative values of their real dielectric function between their LO and TO modes, meaning they functionally behave as optical metals in this region, resulting in high reflectance of the incident infrared light.^69^ This surface phonon polariton enhancement of the near-field signal (which is similar to surface plasmon polaritons in metals) has been observed with n-SNOM and nano-FTIR characterization in polar materials like silicon carbide,^40^ amorphous SiO_2_,^81^ boron nitride,^83^ and SrTiO_3_.^80^ Typically, the result is a broad peak between the LO and TO modes and significant enhancement of the amplitude relative to the reference material (i.e., Au/Si). For instance, a bulk single crystal of SrTiO_3_ has a TO mode at 550 cm^–1^, whereas the corresponding SINS amplitude peak is at 680 cm^–1^ and a near-field enhancement of 3.5 relative to gold, indicating the excitation of surface phonon polaritons.^80^ LiF is also a strong oscillator with negative values of the real part of the complex dielectric function in the 650–312 cm^–1^ range (Figure S6), so the observed behavior for the thickest 445 nm LiF sample is consistent with previous SINS data and means that the peak at 379 cm^–1^ likely corresponds to the excitation of surface phonon polaritons at the LiF/N_2_ interface. Further supporting this conclusion is the 4–6.5× stronger amplitude peak intensity of the LiF thin films relative to the Si reference, indicating a significant near-field enhancement characteristic of the excitation of the surface phonon polaritons in polar solids (Figure S7).

As the thickness of the LiF film decreases, the SINS LiF peak becomes weaker and broader and shifts to higher wavenumbers to reach 572 cm^–1^ for the 10 nm LiF sample on a Si wafer (Figure 2c). This behavior is also consistent with previous work investigating the thickness dependence of amorphous SiO_2_ films from 2 to 300 nm, which showed a significant decrease in the amplitude and shift to the LO frequency with decreasing thickness.^81^ We verified this dependence by collecting and then comparing the behavior of SINS spectra of 20 and 300 nm amorphous SiO_2_ films with the 10 and 228 nm LiF films (Figure S8), which show that the IR absorption peak broadens and shifts toward the LO mode in a similar manner as the thickness of SiO_2_ and LiF films increases. This is an important observation because it suggests that the observed IR spectral behavior is general for polar solid thin films and that the thickness of such thin layers can be estimated based on the magnitude of the peak shift.

LiF crystallinity and domain size are other important parameters for the Li-ion conductivity in the SEI layer.^13,14^ HRTEM was used to determine the structure and morphology of the LiF thin films (Figure 2d,e) to assess a possible relationship to the FTIR spectra. The 137 nm film (Figure 2d) appears to be predominately amorphous with some small <5 nm nanocrystalline domains, whereas the 228 nm film (Figure 2e) displays distinct ca. 5 nm nanocrystalline clusters. The effects of LiF crystallinity and the film thickness on the FTIR spectra are difficult to decouple in this particular case as the thinner LiF film (137 nm) is more disordered than the thicker film (228 nm), which is consistent with HRTEM characterization of sputtered LiF thin films previously reported.^14^ In general, the FTIR spectrum of amorphous vs crystalline materials results in a broadening of absorption bands as the lack of symmetry relaxes the selection rules for the excitation of vibrational modes.^85−87^ However, considering that amorphous SiO_2_ shows a thickness effect in nano-FTIR as described earlier^81^ and the fact that the 228 nm LiF thin film is still very disordered, we conclude that thickness is a more dominant factor in the observed SINS spectra as compared to the differences in crystallinity. This is particularly important because LiF in the SEI tends to be either nanocrystalline or amorphous,^18,35,88^ and the thermally evaporated LiF films may serve as good references.

Overall, the FTIR spectra of the LiF model samples depend on the form factor of LiF and the type of the IR probing mechanism (ATR vs SINS). ATR-FTIR generally agrees well with SINS spectra for organic/molecular compounds (weak oscillators).^30,41^ However, strong oscillators, like LiF, can have significantly different behavior due to (i) their large infrared cross section that can support surface phonon polaritons at certain interfaces (e.g., N_2_/dielectric interfaces) at frequencies where the real part of the dielectric function is negative, and (ii) because the SINS technique is a noncontact method that characterizes the N_2_/material interface. Thus, appropriate references are crucial, especially when probing with SINS for strongly IR resonant polar materials like LiF, LiH, and Li_2_O that each have appreciable splitting between their TO and LO modes, which is an indicator of a strong IR oscillator (Table S1). The need for appropriate references is even more critical when aiming to characterize such materials within complex chemical environments such as the SEI.

### 2.2 SINS Characterization of SEI on a Cu Electrode

To demonstrate the applicability of the proposed form factors in real electrochemical systems, we probed LiF with SINS in the SEI formed on a sputtered Cu thin-film electrode in a 1.2 M LiPF_6_, ethylene carbonate:ethyl methyl carbonate (3:7 wt %) electrolyte. Cu was chosen as a model electrode due to its role as a current collector for Li-ion anodes and the demonstrated ability to preferentially form LiF over organic compounds due to the electrocatalytic reduction of HF in LiPF_6_ electrolytes.^15−19^

**Figure 3:**
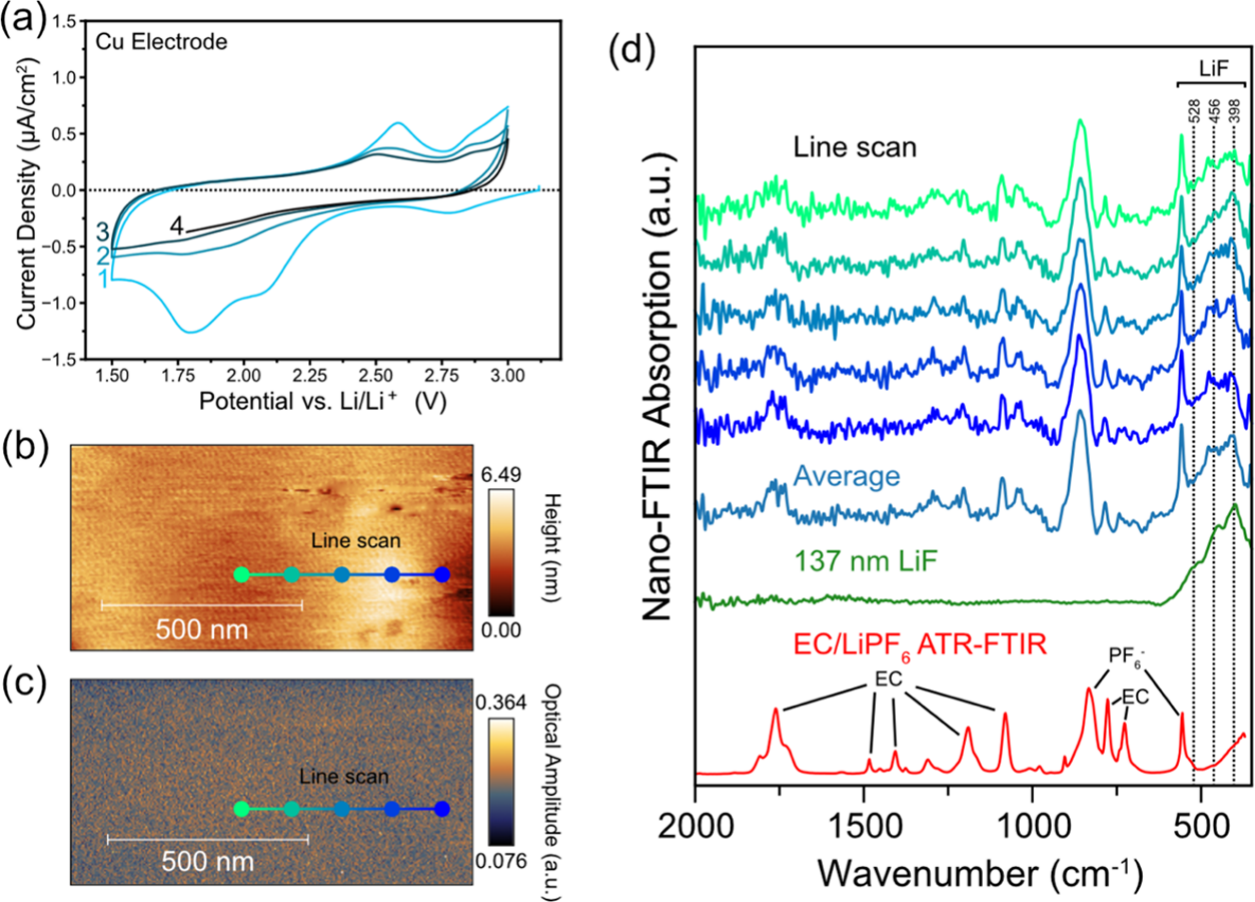
(a) Four initial cyclic voltammograms of a Cu electrode at 0.1 mVs^–1^ between 1.5 and 3.0 V. (b) AFM topography image (1 × 0.5 μm, 3.90 nm/pixel) and (c) IR white-light image of the Cu electrode after the CV cycles. (d) SINS absorption spectra at points shown in the topography and IRwhite-light images along with the average, SINS absorption spectra of the 137 nm LiF thin-film reference and the ATR-FTIR spectrum of the dried residual electrolyte (EC/LiPF_6_).

Figure 3a shows 4 initial CV cycles of the Cu electrode between 1.5 and 3.0 V. During the cycling, the current density of the cathodic and anodic scans decreases gradually, indicating that electrode surface passivation is occurring. The first cycle scan shows a cathodic peak at 1.80 V corresponding to the electrocatalytic reaction of HF to form LiF and H_2_, which matches well the results for a Cu electrode reported by Strmcnik et al.^19^ During the anodic scans, there is a peak at 2.5 V, which also decreases with cycling. It could be directly related to the surface CuO/Li_2_O conversion reaction.^89^ Interestingly, a somewhat similar redox behavior was also observed on a Pt electrode after electrochemical HF removal from carbonate-based LiPF_6_-containing Li-ion battery electrolytes.^90^Figure 3b,c shows the topography and IR white light image of the surface film after the 4 CV cycles, respectively. They reveal a relatively smooth surface with a consistent and uniform IR reflectivity over the scanned area, which indicates chemical homogeneity. This observation is further supported by a series of SINS local spectra (Figure 3d) over the 500 nm line scan, which show similar spectral features, i.e., peak intensity ratios and positions, albeit with some subtle differences that could represent some surface heterogeneity at the nanoscale. The 2000–550 cm^–1^ region shows a variety of peaks, which can be mainly attributed to vibrational modes of the EC and PF_6_^–^ from the residual electrolyte^59,91^ and traces of its organic decomposition products. However, in the 600–350 cm^–1^ region, there is a distinct difference in absorption compared to the electrolyte reference, with a broad feature peaking at ca. 398 cm^–1^.

The spectral features in the 600–350 cm^–1^ region match well with the SINS spectra of the 137 nm evaporated thin film of LiF, which confirms the presence of LiF on the surface of the Cu electrode after cycling. However, the thickness of the SEI layer on the Cu electrode does not exceed a few tens of nanometers, including the LiF sublayer that is expected to be just a few nanometers thick.^18,19^ In fact, 8.5 mC/cm^2^ cathodic charge that was consumed during the 4 CV cycles would produce an 8.6-nm-thick LiF layer over the entire Cu electrode. Moreover, the nano-FTIR amplitude of LiF in the 137 nm LiF film is ca. 10x bigger than in the SEI film on the Cu electrode (Figure S9), which is consistent with the expectation of a weaker near-field response for a thinner film.^52^ It is also possible that the residual electrolyte and other SEI components may screen LiF and impede the near-field coupling with the LiF and the probe. In fact, surface modes are well known to be sensitive to the presence of an overlayer,^71^ and this could potentially explain the discrepancy in the spectral response as the thin LiF layer on the cycled Cu should resemble more the nano-FTIR spectrum of the 10 nm LiF reference thin film (Figure 2c). Due to the local sensitivity of the near-field interaction, the resulting SINS spectra can be altered by small changes to the surrounding chemical environment, which can lead to challenges in the data interpretation and may require numerical simulations to support experimental findings.^52,83^ Simulations of how nano-FTIR spectra of LiF respond to different overlayers and environments found in the SEI will be the subject of future work.

**Figure 4:**
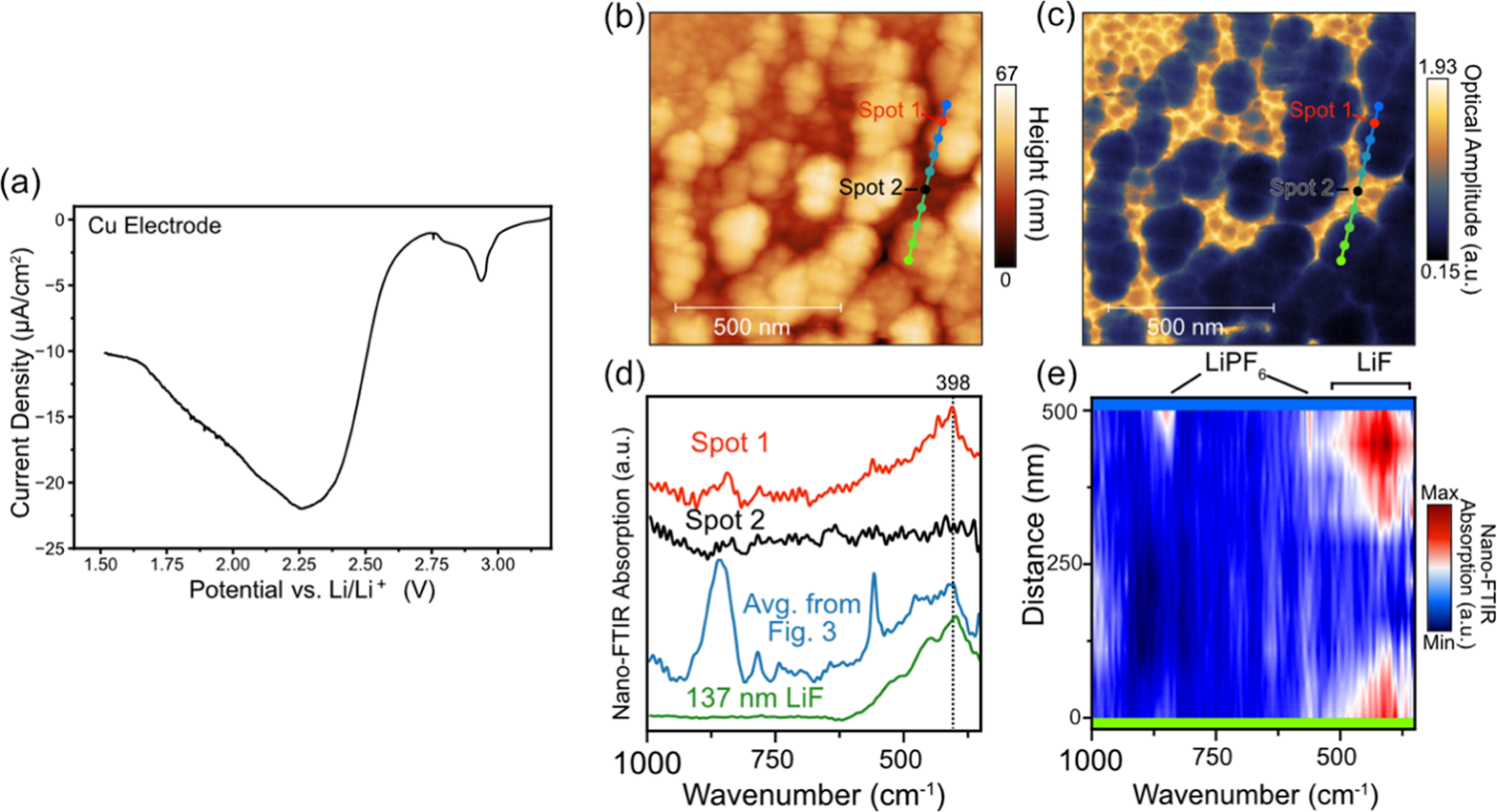
(a) LSV of the Cu electrode to 1.5 V at 0.1 mV/s in the pouch exposed to air before assembly to introduce more water and promote more LiF formation. (b) AFM topography (1 × 1 μm, 3.90 nm/pixel) and (c) IR white-light image of the Cu electrode after the LSV to 1.5 V. (d) SINS absorption spectra at spots 1 and 2 and the reference spectrum of the 137 nm thick LiF on Cu. (e) SINS spectral intensity map along the 500 nm line scan shown in panels (b) and (c).

A similar control experiment was performed with the Cu electrode at higher water and HF contents in the cell. The pouch used to assemble the cell was left under ambient conditions before being transferred to a glovebox and used to assemble a cell. The purpose of this was to introduce more H_2_O (which was absorbed into the pouch) onto the electrolyte, which will react to form more HF by hydrolyzing the PF_5_ molecules.^19^ The larger supply of HF increases the amount of LiF forming on the surface and results in a thicker LiF layer. Figure 4a shows the LSV to 1.5 V of the Cu electrode in the pouch exposed to air before assembly. In good agreement with Strmchnik et al.,^19^ the magnitude of the cathodic peak at 1.8 V increases by a factor of 20× at higher HF concentration in the electrolyte (Figure 3a).

Figure 4b,c shows the AFM topography and white IR light images of the surface of the Cu electrode after the LSV to 1.5 V. In contrast to the cycled Cu electrode described above, the surface is much rougher and heterogeneous in chemical composition. The white IR light image shows a striking difference between the higher and lower areas, suggesting the presence of materials with dissimilar optical properties. Careful inspection of the SINS spectrum at spot 1, which has low IR reflectance (Figure 4d), reveals that the spectrum resembles again the 137 nm LiF thin-film sample (Figure 2c) with a broad absorption feature beginning at around 625 cm^–1^ and reaching a maximum at 398 cm^–1^. Based on the expected presence of LiF on the Cu surface and the good match with the reference, we assign the broad spectrum to LiF particles that have grown on the Cu surface. The AFM height profile across the line scan (Figure S10) indicates that the thickness of the LiF layer on the Cu electrode is 24 nm in this case, which is quite different than the theoretical thickness of a LiF layer ca. 181 nm, assuming all of the charge went to form LiF. This large discrepancy suggests other electrolyte reduction reactions, which could yield soluble products that quickly dissolve in the electrolyte.

Spot 2, however, which lies in the more reflective region of the white light image, shows no discernible IR bands at frequencies <1000 cm^–1^. A comparison of the nano-FTIR amplitudes of the line scan (Figure S11) shows that the intensity in the more reflective region is ca. 1.75 and corresponds to a material more IR reflective than the Si reference. This strongly supports the view that the regions showing high white light intensity (Figure 4c) represent a Cu surface with a very thin SEI layer, which results in a higher IR reflectance than Si. This IR signal intensity pattern in the 600 to 350 cm^–1^ range matches the morphology and white IR light images, as demonstrated by a high-resolution 500 nm line scan (Figure 4e).

While the primary purpose of these two experiments on the Cu electrode was to demonstrate the nanoscale detection of LiF, they provided some insights into the mechanism of LiF nucleation and growth under the two different conditions examined. During CV cycling of the Cu electrode, the decreasing cathodic current density indicates that passivation of Cu takes place mainly during the first cathodic scan. The dense and uniform LiF film appears to cover the entire surface, which is a common characteristic of effective passivation layers. In contrast, the Cu electrode polarized cathodically in the electrolyte with higher HF content showed a rough, highly heterogeneous, and thicker LiF film with large LiF clusters scattered on the Cu surface just after a single LSV scan to 1.50 V. The nucleation and growth of LiF on the electrode surface depends on a variety of factors, including electrode potential, electrode surface structure,^92^ concentration/diffusion of LiF forming reactants (e.g., HF), and solubility of LiF. The HF concentration not only depends on the amount of water but also the presence of linear or cyclic carbonates in the electrolyte.^17^ It is also possible that the concentration and diffusion of PF_6_^–^, another source of LiF,^18,93^ at the interface can also influence the resulting LiF morphology. From the results presented here, we suggest that the HF concentration in the electrolyte is a factor that can influence LiF morphology and associated passivating properties in SEI layers, although more detailed experiments and comparisons will be needed to confirm this.

### 2.3 SINS of the SEI Layer on Si-Based Anodes

**Figure 5:**
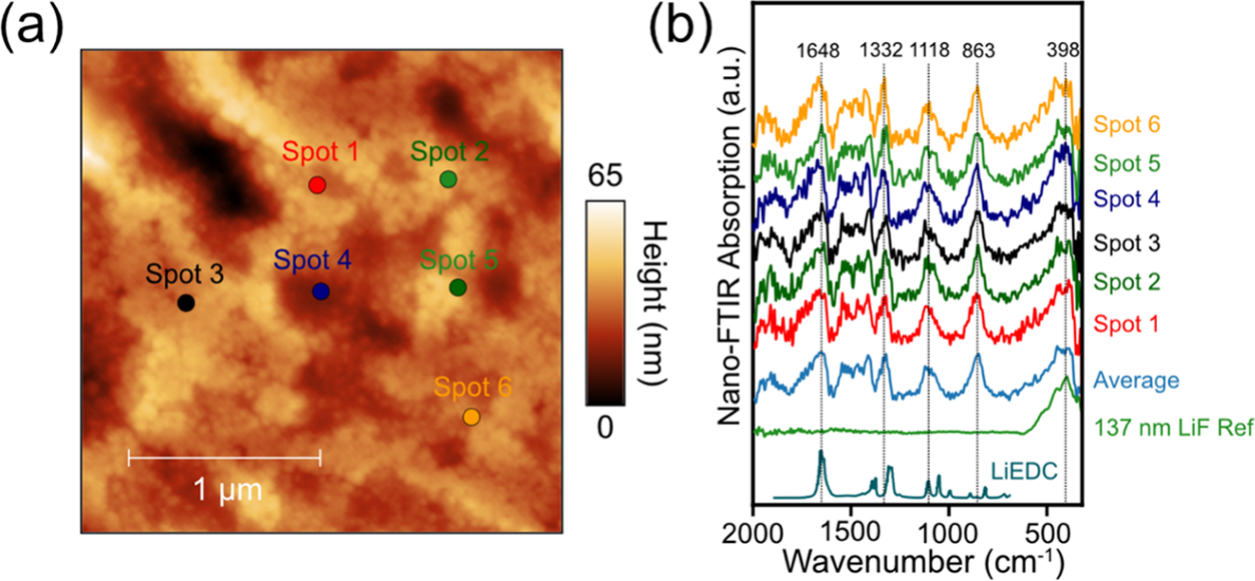
(a) AFM topography (2.5 × 2.5 μm, 12.5 nm/pixel) of the 50 nm thin-film amorphous Si electrode after LSV to 0.05 V at 0.1 mV/s. (b) SINS absorption spectra at spots 1–6 together with LiF thin-film reference spectra and lithium ethylene decarbonate (LiEDC) reference spectra.^24^

Next, we characterized the SEI on the surface of a model thin-film Si electrode to investigate the structure and composition of the SEI layer after a single cathodic scan. The AFM topography image and SINS local spectra of a 50 nm amorphous Si electrode after a linear potential scan from the open-circuit potential to 0.05 V (LSV in Figure S12) are shown in Figure 5. The AFM topography (Figure 5a) shows the densely packed SEI layer conformal to the surface of the Li_*x*_Si thin film.^30^ The SINS local spectra (Figure 5b) at spots 1–6 show nearly identical spectral features, suggesting that the composition of the SEI layer is relatively homogeneous on these length scales. A prominent peak at 1648 cm^–1^ is characteristic of Li alkyl carbonates,^24,59^ and the presence of a peak at around 863 cm^–1^ represents the various P–F- and P–O–F-related decomposition products derived from the PF_6_^–^ anion.^59^ IR absorption peaks at 1332 and 1118 cm^–1^ are related to the C–O stretching mode of lithium ethylene decarbonate (LiEDC), which is consistent with our findings in our previous work.^25,59^ A broad peak at around 398 cm^–1^ matches well with the 137 nm LiF reference, suggesting that LiF is present in all spots 1–6, along with organic decomposition products.

LiF being observed together with organic decomposition products at the nanometer scale has two major implications for evaluating the structure of the SEI layer on the Si thin-film electrode. Since the probing area of SINS is around 300 nm^2^ and the signal is collected from the entire depth of the SEI layer film ca. 50 nm,^33^ the SINS spectra indicate that LiF and organic species, i.e., LiEDC, alkyl carbonates are evenly distributed in each of the probing areas, and most likely throughout the entire SEI layer. Second, this result supports our initial model of the SEI presented in our previous work, where the SEI layer was comprised of an organic matrix with LiF particles distributed throughout, which could form because of PF_6_^–^ infiltration and decomposition through the organic components.^30^

To further investigate the key factors related to the surface passivity of Li-ion anodes, SINS was used to probe the SEI layer chemistry on the Si_40_Al_50_Fe_10_ metallic glass electrode after the single formation charge–discharge cycle. Metallic glasses constitute a promising class of Si-based electrode materials that show cycle life, higher interfacial stability, and improved calendar life compared to pure Si electrodes.^94^ The transition-metal surface constituents and the amorphous structure of the glasses can alter the kinetics of the electrolyte decomposition reactions, resulting in different distributions of products and local film structures. The initial XPS results suggest that the surface of the amorphous metallic phase forms a more inorganic-rich (LiF, F–P–O) SEI layer as opposed to pure Si, which forms a more solvent-derived, organic SEI layer comprised of LiEDC and alkyl carbonate oligomers.^94^ Furthermore, the splat quenched foil is compatible with model Cu and Si electrodes used in this study, free of binders and carbon additives present in composite anodes, which can significantly complicate the interpretation. Figure S13 shows the first galvanostatic formation cycle of the Si_40_Al_50_Fe_10_ electrode at C/25 between 1.5 and 0.05 V.

**Figure 6:**
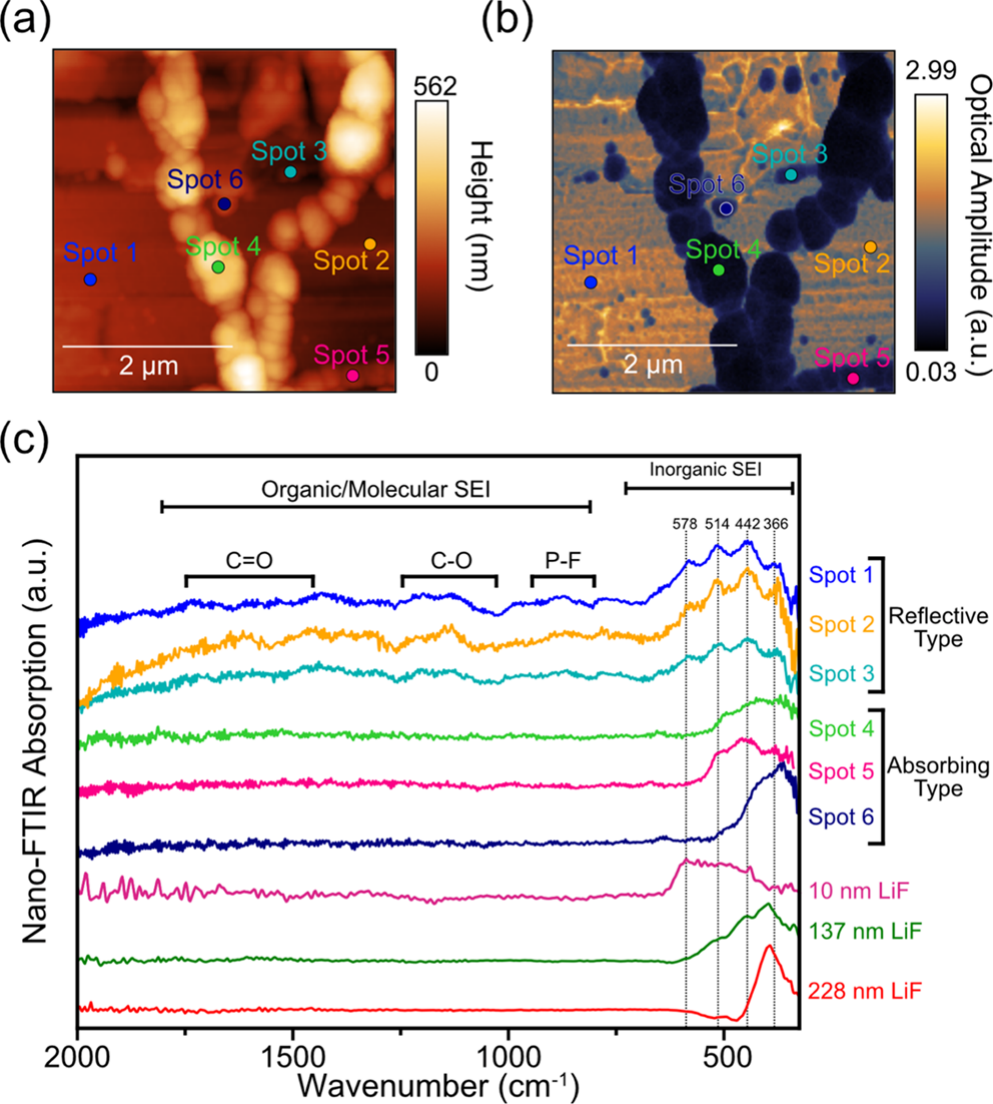
(a) AFM topography (4 × 4 μm, 23.5 nm/pixel) and (b) IR white-light image of the Si_40_Al_50_Fe_10_ electrode after the 1 formation cycle at C/25. (c) SINS absorption spectra recorded at different locations of the surface film (spots 1–6) together with LiF thin-film reference spectra.^42^

Figure 6a shows the surface topography image of the Si_40_Al_50_Fe_10_ electrode after the first formation cycle at C/25. The surface chains of large ca. 100–200 nm particles on a relatively flat, densely packed sublayer are observed. In the corresponding white-light image (Figure 6b), these particles strongly absorb the incoming IR light, somewhat similar to the image observed on the Cu electrode in the presence of water (Figure 4c). In contrast, the smoother sublayer is more IR reflective.

SINS spectra (Figure 6c) from the IR reflective sublayer (spots 1–3) show a broad maximum, which consists of IR bands at 578, 514, 442, and 366 cm^–1^ from LiF and a series of weak bands in the region from 2000 to 700 cm^–1^, from organic/molecular SEI compounds, which is consistent with a thin predominantly LiF film with traces of organic phases similar to the SEI layer observed on the Cu electrode. These higher wavenumber features broad and roughly correlate with the regions corresponding to symmetric and asymmetric vibrations of C=O, C–O, and P–F bonds.^30^ The LiF bands appear to be a combination of spectral features of the 10 and 137 nm reference LiF thin films. A higher amplitude and a lower relative height of the LiF peaks would also indicate a thinner LiF film made of densely packed nanoparticles.

The SINS spectra of IR absorbing large particles (spots 4–6) show the same bands characteristic for LiF, but their intensity varies greatly with the location. The large size of the absorbing LiF particles (100–200 nm) is similar to the LiF thin-film reference spectra for 137 and 228 thicknesses (Figures 2c and S14). Furthermore, comparing the magnitude of the amplitudes (Figure S15) of the 6 spots, the absorbing spots have a much lower amplitude than the reflective sublayer, suggesting that the surface probed in the absorbing spots comprises a material with a lower electronic conductivity, consistent with the presence of a thick inorganic layer.^48^ Interestingly, the spectral response from the large LiF particles lacks any distinct features corresponding to organic electrolyte decomposition compounds.

Based on these results, we can make some general observations about the SEI layer formed after 1 formation cycle at the Si_40_Al_50_Fe_10_ metallic glass electrode. First, there are two distinct types of LiF morphology present on the surface The inner dense layer consists of a fine, densely packed LiF nanoparticle scaffold that provides structural support for polymeric compounds. Notably, this mixed inorganic/organic sublayer appears to have a greater fraction of LiF compared to the SEI layer on the amorphous Si thin film (Figure 5b). The clusters of large particles on top of the dense film are comprised of solely inorganic phases, most likely LiF, based on the good agreement with our reference spectra. However, it is possible that Li_2_O and LiH could also show absorption in a similar range and be present in these regions. We suspect it is likely that the thinner SEI provides the path of least resistance for Li^+^ transport as compared with the thick 100–300 nm particles of LiF. Interestingly, such a dual SEI structure is similar to what was observed in our previous IR imaging study of the SEI on the highly oriented pyrolytic graphitic (HOPG) electrodes where larger precipitates were present over a thinner conformal SEI layer.^56^

This dual SEI morphology points to a mechanism by which small and large LiF precipitates nucleate and coalesce on the surface of the electrode simultaneously. PF_6_^–^ anions must decompose to form large LiF particles; however, the mechanism of this transformation is not completely clear. It is possible that the organic components of the SEI allow for PF_6_^–^ transport into the reactive electrode surface and allow for PF_6_^–^ decomposition to LiF or other soluble species, leading to the growth of 100′s of nm of LiF. Spotte-Smith and Petrocelli et al. demonstrated with computation that the chemical reaction of Li_2_CO_3_ with PF_5_ could lead to the formation of POF_3_ with solid LiF as a byproduct of several reaction steps.^95^ Further computational results have also suggested that LiF clusters can form and then migrate to the surface and that this process depends on the surface termination of the electrode.^92^ Cao et al. also demonstrated with computation that existing LiF nuclei can result in accelerated PF_6_^–^ decomposition as precipitating LiF on a surface is energetically favorable.^18^ Furthermore, LiF solubility and the dissolution rate in the electrolyte are also likely to play a factor for coarsening of small LiF nuclei into larger particles.^21^

## 3 Conclusions

We investigated the use of SINS to characterize the nanoscale structure and distribution of LiF in the SEI on Li-ion anodes. Bulk and nanoscale LiF model samples reveal sensitivity to LiF surface phonon modes and the chemical environment of LiF. The bulk LiF model samples (powder, pellet, single crystal) demonstrated a significantly different infrared absorption than deposited thin films of LiF (137–445 nm), suggesting that bulk reference samples of the SEI layer components that are strong oscillators are not applicable to the nanoscale domains of the SEI. This discrepancy is attributed to the excitation of surface phonon polaritons, which originate from size and interface effects and contribute to the infrared absorption spectrum more than the bulk.

Therefore, we conclude that when probing the SEI layer with infrared techniques, surface phonon modes play a significant role in the resulting infrared spectrum for Li-inorganic phases, which are strong oscillators (e.g., LiF, Li_2_O, LiH). As a result, we believe it is crucial to have model systems that match with the SEI layer length scale (e.g., thin films, nanoparticles) to accurately assign the spectral features obtained from SEI layers. Finally, we emphasize that the infrared characterization method (e.g., ATR vs SINS) will also play an important role in determining the spectra features due to the length scale of excitation and different interfaces that are probed.

Local SINS spectra of the SEI layer on Cu, Si, and Si_40_Al_50_Fe_10_ metallic glass electrodes reveal the presence and various distribution patterns of LiF. We find that spectra of all of the electrode surfaces featured broad absorption in the 600–325 cm^–1^ region, which matches well with the surface phonon mode observed in the spectra of model LiF thin films. Most notably, each electrode displayed a different spatial distribution of the observed LiF showcasing the nuance and heterogeneity of the SEI at the nanoscale, information that is often overlooked with conventional characterization methods. For instance, the two Cu electrodes investigated had LiF present on the surface but with different distributions and thicknesses, which could then be correlated to the passivity based on voltammetry. The surface of the thin-film Si electrode after first lithiation showed a homogeneous SEI layer comprised of a nanoscale mixture of LiEDC and LiF as both were observed in the same nanoscale area. In contrast, the SEI layer of a metallic glass electrode, investigated after 1 formation cycle, shows several regions of distinct spectra, which we identify as a thin mixed organic/inorganic SEI and a thicker inorganic SEI. Both regions on the metallic glass display slightly different absorption in the region from 600 to 325 cm ^–1^, which is attributed to LiF of different thicknesses. These different SEI morphologies and chemistries show the nuanced structure of the SEI on the nanometer scale and how different electrodes and electrochemical procedures can produce various ratios of organic and inorganic products at the surface.

Overall, we demonstrate how SINS can be utilized to characterize the nanoscale position and thickness of LiF in the SEI and provide information about the heterogeneity of the SEI layer. The importance of the SINS characterization is that we can assess the nanoscale morphology and topology of the organic and inorganic components of the SEI layer over the micrometer-length scale with a noninvasive, low-photon-energy technique. This provides opportunities to gain insights into how the concentration and mass transport of LiF reactants connect to its deposited morphology and subsequent passivation, which hitherto has been inaccessible with routine characterization methods. Future work dedicated to understanding these relationships in detail will be fruitful for the optimization of the inorganic phase content and morphology in the SEI layer.

## 4 Materials and Methods

### 4.1 LiF Reference Samples

The LiF pellet was fabricated from LiF powder (99.995%, precipitated, Aldrich) by isostatic pressing (24 MPa), followed by sintering at 650 °C for 2 h in air. The resulting LiF pellet was then polished with a 2600 grit SiC sandpaper. LiF single crystals were purchased from MTI (LiF single crystal for evaporation, purity >99.995%, 5 mm × 5 mm× 5 mm as cut) and used as the evaporation source for the LiF thin films. LiF thin films were deposited on a Cu or Si substrate at room temperature via thermal evaporation using a baffled box source (Baffled Box, 5 g, SM-10, RD Mathis Company). The deposition rate was 1–3 Å/s at a base pressure of 2.9 × 10^–6^ Torr, with the thickness measured with FIB-SEM (Helios G4 UX, FEI).

### 4.2 Model Electrodes: Cu, Thin-Film Si, and Si_40_Al_50_Fe_10_

The Cu electrodes were fabricated with sequential DC sputtering onto a 1/2 in. diameter quartz wafer substrate (University Wafer, U01-210714-1: Fused Silica JGS2). First, a thin layer of Ti (30 nm) was DC sputtered (3 × 3” TORUS Mag Keeper sputter guns) as an adhesion layer, followed by deposition of a 1.0-μm-thick Cu layer with a low RMS surface roughness of 3.7 nm.^30^ Notably, it has been shown that Cu oxide on the surface can affect the electrochemistry in Li-ion battery electrolytes.^96^ No attempts were made to remove Cu oxide from the surface, although we suspect that the oxide layer was minimal as the Cu electrodes were stored in an argon glovebox after deposition and prior to cell assembly. The 50 nm thin-film amorphous Si electrode was fabricated with DC sputtering in a similar manner to our previous work.^30^

The Si_40_Al_50_Fe_10_ amorphous glass foil was produced with a splat quencher (Ultra Rapid Quenching, Edmund Buhler GmbH). Prior to splat quenching, the parent alloy was prepared with Si (Sigma-Aldrich, 99.95%), Al (Alfa Aesar, 99.99%), and Fe (Sigma-Aldrich, 99.95%) via arc melting (SA-200, MRF) under an Ar atmosphere. Twenty milligrams of the alloy was placed inside a boron nitrite crucible in the splat quencher chamber, followed by 3 cycles of purging with Ar. The chamber was evacuated to a pressure <10^–5^ mbar and then filled with Ar to 600 mbar. The alloy was then magnetically levitated inside the induction coil and melted. The molten alloy droplet was then released from the coil and splattered between two Cu pistons. The quenching rate of this process is 10^5^–10^6^ K s^–1^.

### 4.3 Electrochemical Characterization

The Cu, Si thin-film, and Si_40_Al_50_Fe_10_ electrodes were evaluated electrochemically in two-electrode pouch cells, using a Li metal foil (13 mm diameter,1.0 mm thickness, MTI Corporation) as the counter and the reference electrode with a Celgard 2635 separator (20 mm diameter). The pouch cells were assembled in poly foil bags (Sigma-Aldrich, Z183385) and cut into 5.5 cm × 6 cm sections. Ni tabs were used to provide electronic wiring to the electrodes, and each cell was heat-sealed with adhesive polymer tape (MTI Corporation, EQ-PLiB-NTA4). The cells were filled with 10 μL of a Gen 2 electrolyte (1.2 M LiPF_6_ in EC: EMC 3:7 wt %, Tomiyama Chemicals, Japan). All cell assembly was performed in an Ar-filled glovebox with O_2_ and H_2_O below 0.1 ppm. After cell assembly, a light mechanical pressure was applied to the cell with a clamp. Electrochemical cycling with cyclic voltammetry or galvanostatic methods was performed with a Biologic VMP3 potentiostat. For characterization, the cells were disassembled in the glovebox, and the electrodes were characterized without washing.

### 4.4 Transmission Electron Microscopy

The local nanostructure of LiF was investigated with HRTEM (Titan X 60-300, FEI), operating at 200 kV. The sample was thinned by FIB (Ga ion beam on Helios G4 UX. FEI) after a 1 μm Pt coating for surface protection. The structural analysis, including high-angle annular dark-field (HAADF)-STEM, was conducted using a probe-side aberration-corrected TEM. The elemental mapping was collected from EDS (Bruker) equipped within the TEM at 300 kV.

### 4.5 AFM and Synchrotron Infrared Nanospectrosocpy

Synchrotron infrared nanospectroscopy and AFM measurements were conducted with a Neaspec neaSCOPE microscope housed at the Advanced Light Source (ALS) at beamline 2.4 using PtIr-coated AFM probes from Neaspec. Supplementary nano-FTIR with the lab broadband lasers (Figure 1b) was conducted with a similar Neaspec n-SNOM microscope housed in our lab and used similarly to our previous work.^30^ Both microscopes were housed in an environmental chamber continuously purged with N_2_, and measurements were performed at O_2_ < 20 ppm (PureAire, Trace Oxygen Analyzer 0–1000 ppm). AFM topography images were recorded in tapping mode using a tapping amplitude of 50–60 nm. Images were processed, and root-mean-square (RMS) surface roughness was calculated using Gwyddion.^97^

SINS spectra were recorded with a tapping amplitude of 50–60 nm with a spectral resolution of 8 cm^–1^. Near-field IR nanoimages, “white light images,” were collected simultaneously with the AFM topography measurements by fixing the interferometer mirror position to the most intense feature of the interferogram and recording the resulting second harmonic of the optical amplitude value at each spatial pixel. Reference spectra were taken on a polished Si wafer. Spectra were processed using neaPLOT (neaspec). All spectra were demodulated at the *n* = 2 harmonic. It is common in the literature to find either the phase or the imaginary part of the complex nano-FTIR spectrum reported as absorption. This is because in the small angle/phase approximation, the two are mathematically proportional to one another: *z*″ = *A* sin ϕ → z″ ≈ *A*ϕ_small_. In this work, we frequently measure strong resonances marked by significant phase responses, and so we report the second harmonic of the imaginary component of the complex-valued nano-FTIR spectra normalized to Si as nano-FTIR absorption. This is because in these strong resonant cases, the imaginary part most closely matches FTIR absorption databases.^98,99^

### 4.6 X-ray Photoelectron Spectroscopy (XPS)

XPS was performed with a Thermo-Fisher K-Alpha Plus XPS using an Al X-ray source (1.486 eV). A flood gun for charge neutralization was used in all of the experiments. The survey scan was taken from −20 to 1400 eV with a pass energy of 200 eV and an energy spacing of 1.0 eV. The high-resolution spectra were collected with a pass energy of 150 eV and an energy spacing of 0.2 eV. No attempts were made to reference the binding energy of the XPS spectra, which are presented without processing.

### 4.7 ATR-FTIR Spectroscopy

Attenuated total reflectance Fourier transform infrared (ATR-FTIR) spectroscopy measurements were performed inside a N_2_-filled environmental chamber (818GBB/Plaslabs). A Shimadzu IRTracer-100 spectrophotometer outfitted with the single reflection PIKE technologies IRIS ATR sampling accessory equipped with the extended range diamond ATR was used to record ATR–FTIR spectra. The FTIR spectra had a collection range of 4000 cm^–1^–375 cm^–1^ and were averaged over 40 scans with a spectral resolution of 4 cm^–1^.

## Supporting Information Available

LO and TO modes of LiH, LiF, and Li_2_O; FIB-SEM of evaporated LiF thin films of different thicknesses; HAADF image and STEM EDS mapping of the 228-nm-thick LiF thin film; XPS spectra of the 228- and 10-nm-thick LiF thin film; AFM topography image of the 228-nm-thick LiF thin film; real and imaginary dielectric function of LiF; nano-FTIR amplitudes of LiF thin films; comparison of the amplitudes of thin and thick layers of SiO_2_ and LiF; comparison of nano-FTIR amplitudes of the 137 nm LiF thin film and the SEI on the Cu electrode; height profile of the line scan in Figure 4; nano-FTIR amplitudes of the line scan in Figure 4e; LSV of the thin-film a-Si electrode; voltage profile of the formation cycle of the Si_40_Al_50_Fe_10_ metallic glass electrode; height profiles of several particles corresponding to spots 4 and 6 in Figure 6; and nano-FTIR amplitudes of the 6 spots in Figure 6 (PDF)
