# Supplementary material for: Synchrotron Near-Field Infrared Nanospectroscopy and Nanoimaging of Lithium Fluoride in Solid Electrolyte Interphases in Li-Ion Battery Anodes
Source: ACS Nano. 2024 May 24;18(23):15270–83. doi: 10.1021/acsnano.4c04333 (PMC11171761; doi:10.1021/acsnano.4c04333)
Supplement: Supplementary file 1 — nn4c04333_si_001.pdf [file nn4c04333_si_001.pdf]

Supplemental Information for

Synchrotron Near-Field Infrared Nanospectroscopy and Nanoimaging of Lithium Fluoride in Solid Electrolyte Interphases on Li-ion Battery Anodes

Andrew Dopilka[1], Jonathan M. Larson[2,1], Hyungyeon Cha[1,3], Robert Kostecki[1]*

[1]Energy Storage and Distributed Resources Division, Lawrence Berkeley National Laboratory, Berkeley, California 94720, United States

[2]Department of Chemistry and Biochemistry, Baylor University, Waco, Texas 76798, United States

[3] Ulsan Advanced Energy 944 Technology R&D Center, Korea Institute of Energy Research 945 (KIER), Techno-saneop-ro 55 beon-gil, Nam-gu, Ulsan 44776, Republic of Korea

*Email: r_kostecki@lbl.gov

Table S1. Longitudinal Optical (LO) mode and transverse optical (TO) mode frequencies from the literature for LiH, LiF, and Li_2O.

Material	Longitudinal Optical Mode (cm^{-1})	Transverse Optical Mode (cm^{-1})
LiH[1]	1120	592
LiF[2–4]	667	312
Li_2O[5]	737	425

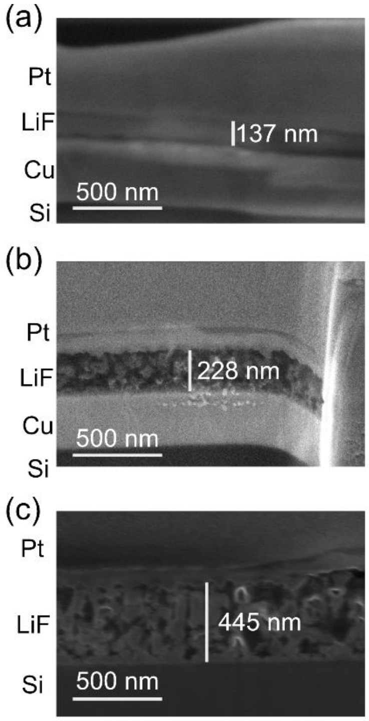

Figure S1. SEM of a FIB cross section of the LiF thin films of different thicknesses that were analyzed in Figure 2.

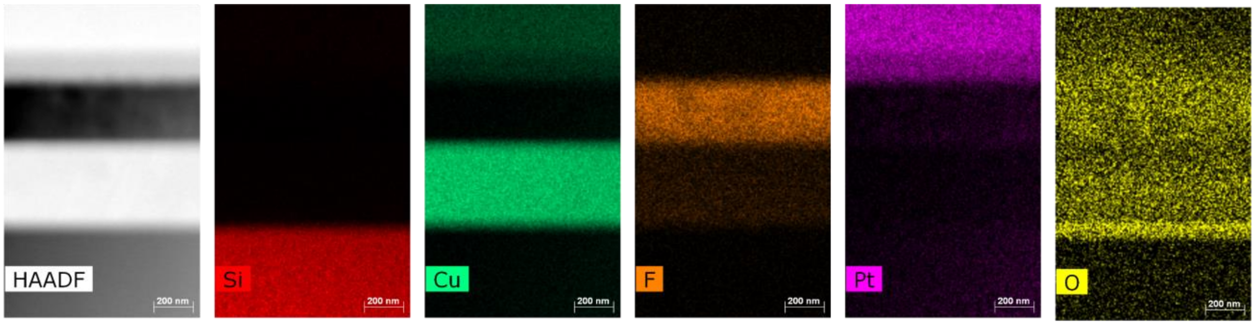

Figure S2. HAADF image and STEM EDS mapping of the cross section of the 228 nm LiF thin film.

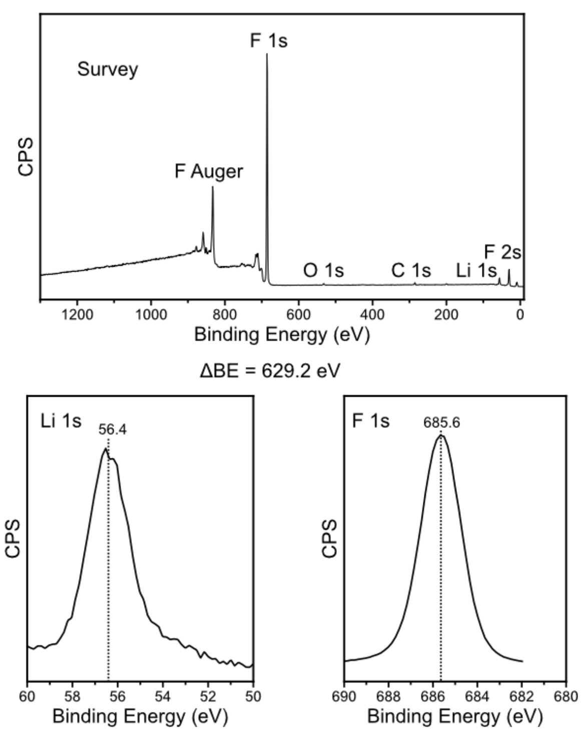

Figure S3. XPS spectra of the evaporated LiF thin film with thickness of 228 nm. Survey spectra is at the top with the Li 1s and F1s at the bottom.

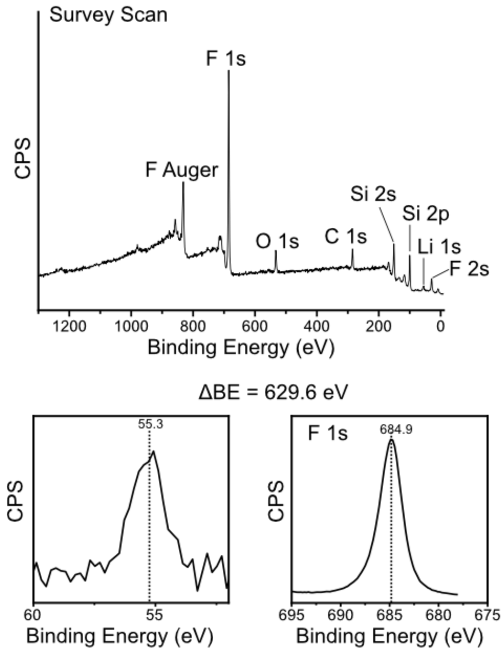

Figure S4. XPS spectra of ca. 10 nm evaporated LiF thin-film on a Si wafer. The thickness estimate is based on the fact that Si underlayer is present in the XPS, so the LiF thickness must be less than the photoelectron escape depth which is around 10 nm.

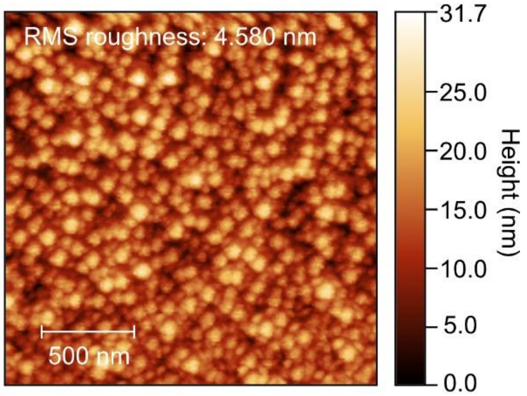

Figure S5. AFM topography image of the 228 nm thick, thin film of evaporated LiF.

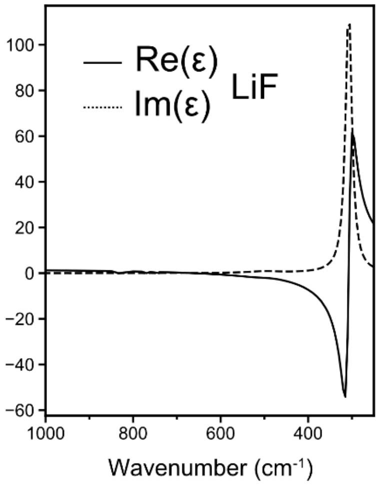

Figure S6. Real and imaginary dielectric function (ε) of LiF from Palik.[6]

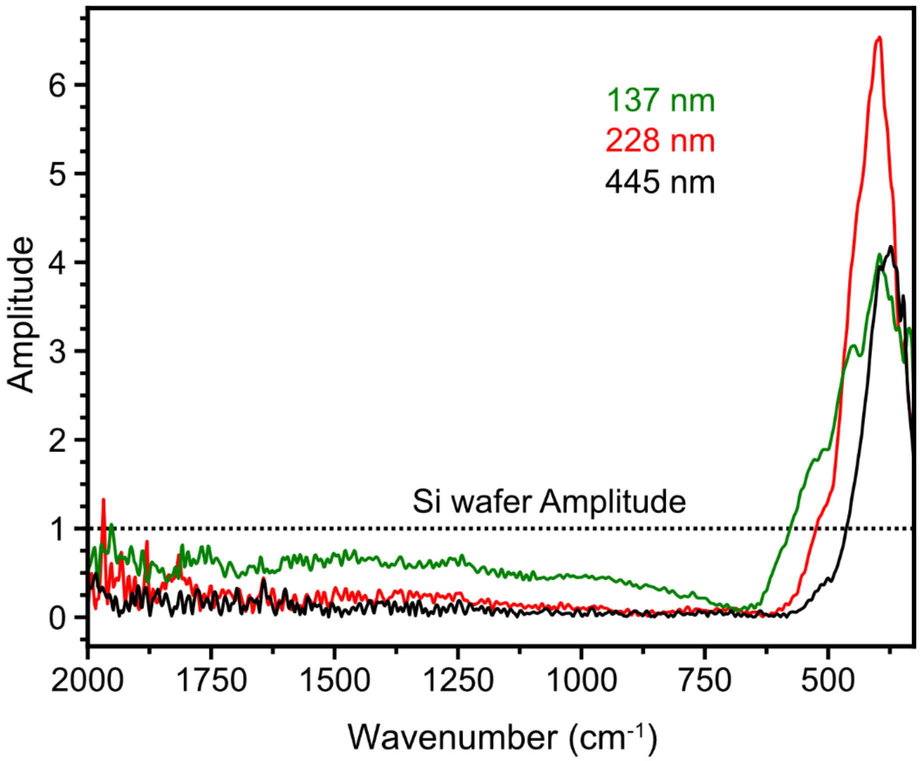

Figure S7. Amplitudes of the 137, 228 and 445 nm thick LiF thin films showing the near-field enhancement (Amplitude > 1) originating for the near-field coupling with the surface phonon polaritons of LiF.

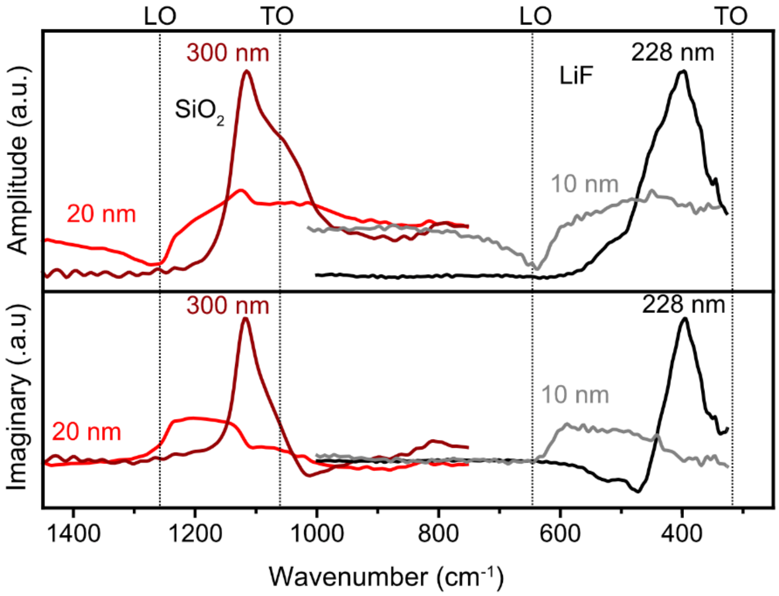

Figure S8. A comparison of SiO_2 and LiF thin films and their thickness dependent spectral shape. The 20 nm SiO_2 spectra was taken from the TGQ1 calibration sample, and the 300 nm was collected from a

thermally grown 300 nm SiO2/Si wafer. The LO and TO modes of SiO_2 from ref[7] and LiF and indicated by the dashed lines. The spectra were normalized to each other to serve for a better comparison of the shape.

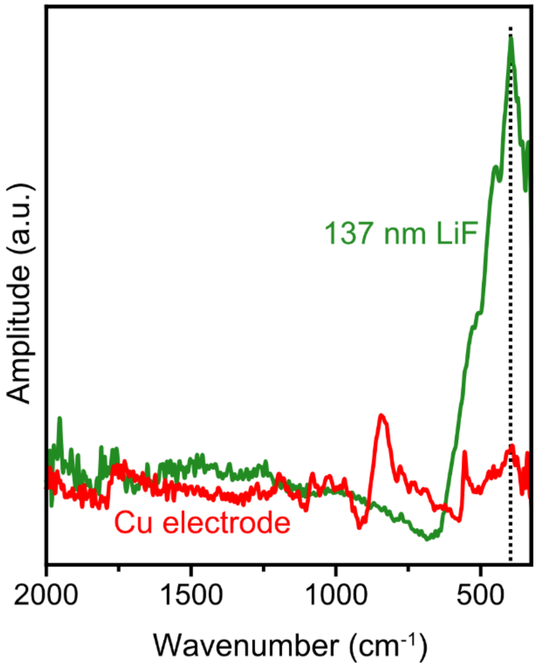

Figure S9. SINS amplitudes are referenced to a Si wafer of the 137 nm LiF reference to the spectra of the Cu electrode in Figure 3. The 137 nm LiF shows a much higher amplitude response compared to the Cu electrode at 400 cm^{-1} suggesting that there is less LiF present on the Cu electrode or less near-field coupling.

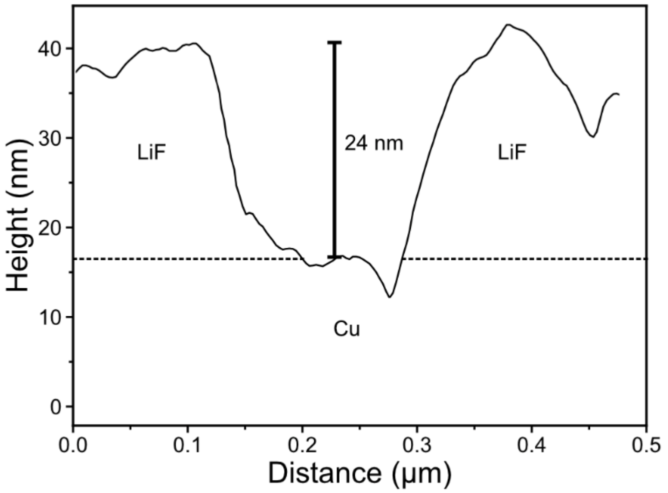

Figure S10. Height profile of the line scan corresponding to Figure 4 showing that the thickness of the LiF is around 24 nm.

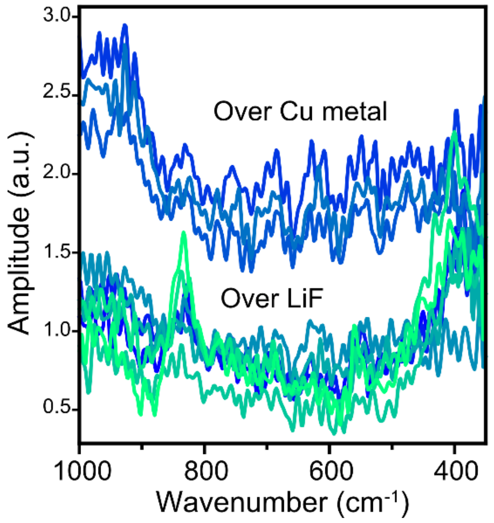

Figure S11. SINS amplitudes referenced to a Si wafer of the line scan shown in Figure 4. The amplitude values above 1.0 when referenced to a Si wafer are indicative of a phase that is more electronically conductive than Si which strongly supports the presence of Cu metal being present in this region. When the probe is over LiF/Cu the amplitude attenuates significantly due to the electronic insulating nature of LiF.

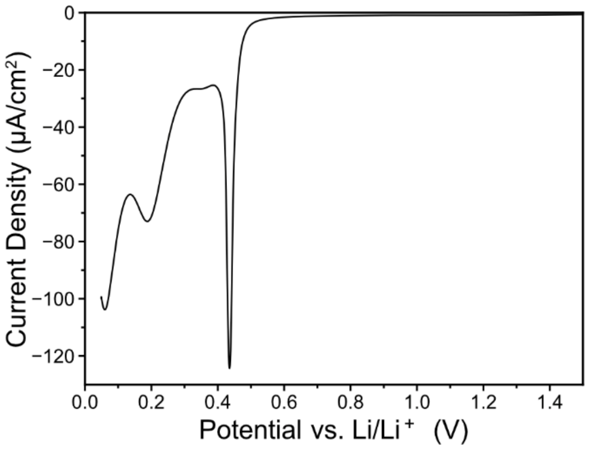

Figure S12. LSV of the thin film a-Si electrode to 0.05 V at 0.01 mV/s.

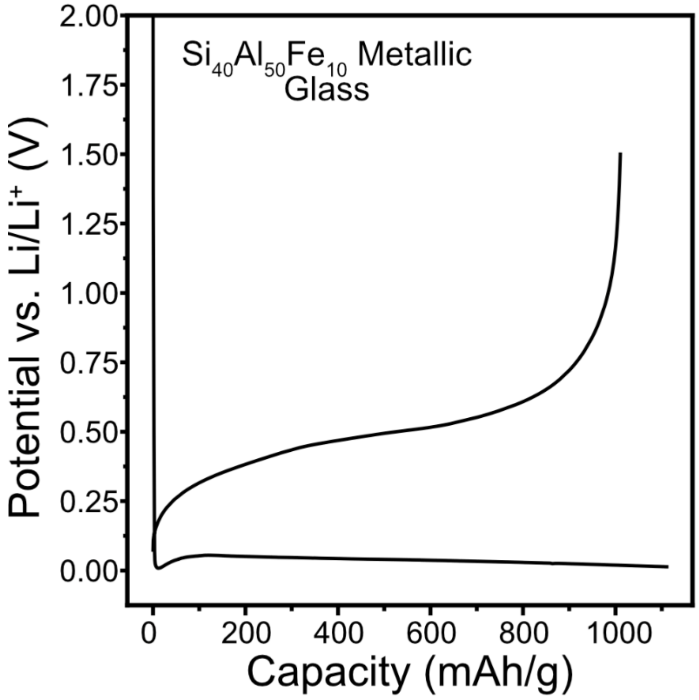

Figure S13. Voltage profile of the formation cycle (from 1.5 to 0.05 V) of the $Si_{40}Al_{50}Fe_{10}$ splat quenched metallic glass electrode at 47 mA/g of Si + Al (0.05 C).

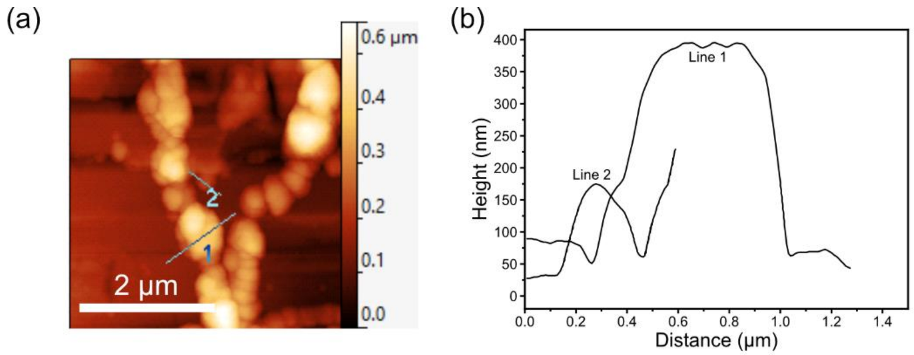

Figure S14. (a) AFM topography of metallic glass after formation cycle with line profiles. (b) Height profile over the areas corresponding to Spot 4 and Spot 6 as seen in Figure 5.

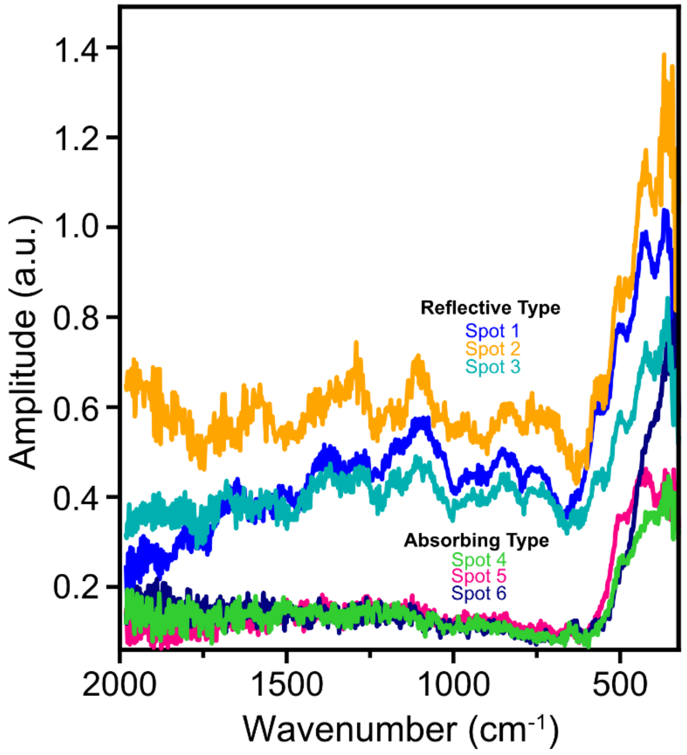

Figure S15. SINS amplitudes referenced to a Si wafer of Spots 1-6 shown in Figure 5. Spots 1-3 show higher amplitude values compared to Spots 4-6 indicating a significantly different interaction with the broadband IR light.

References

(1) Brodsky, M. H.; Burstein, E. Infrared Lattice Vibrations of Single Crystal Lithium Hydride and Some of Its Isotopic Derivations. *J. Phys. Chem. Solids* **1967**, *28* (9), 1655–1668. https://doi.org/10.1016/0022-3697(67)90139-4.

(2) Willett-Gies, T. I.; Nelson, C. M.; Abdallah, L. S.; Zollner, S. Two-Phonon Absorption in LiF and NiO from Infrared Ellipsometry. *Journal of Vacuum Science & Technology A: Vacuum, Surfaces, and Films* **2015**, *33* (6), 061202. https://doi.org/10.1116/1.4927159.

(3) Gottlieb, M. Optical Properties of Lithium Fluoride in the Infrared*. *J. Opt. Soc. Am.* **1960**, *50* (4), 343. https://doi.org/10.1364/josa.50.000343.

(4) Humlíček, J. Infrared Ellipsometry of LiF. *Thin Solid Films* **1998**, *313*, 687–691. https://doi.org/10.1016/s0040-6090(97)00978-4.

(5) T., Osaka; I., Shindo. INFRARED REFLECTIVITY AND RAMAN SCATTERING OF LITHIUM OXIDE SINGLE CRYSTALS. *Solid State Communications* **1984**, *51* (6), 421–424.

(6) Palik, E. D. *Handbook of Optical Constants of Solids*; Elsevier, 1985.

(7) Zhang, L. M.; Andreev, G. O.; Fei, Z.; McLeod, A. S.; Dominguez, G.; Thiemens, M.; Castro-Neto, A. H.; Basov, D. N.; Fogler, M. M. Near-Field Spectroscopy of Silicon Dioxide Thin Films. *Physical Review B - Condensed Matter and Materials Physics* **2012**, *85* (7), 1–8. https://doi.org/10.1103/physrevb.85.075419.
